# N-terminal pro-b-type natriuretic peptide (nt-pro-bnp) as outcome predictor after cardiac surgery (CS)

**DOI:** 10.1186/2197-425X-3-S1-A110

**Published:** 2015-10-01

**Authors:** A Pavalascu, T Arce Arias, ML Jimenez Lizarazu, JM Nuñez Martinez, E Tebar Boti

**Affiliations:** Hospital Universitario Vinalopo, Elche, Spain

## Introduction

Plasma NT-PRO-BNP is a known diagnostic and prognostic heart failure biomarker, correlate with ventricular wall stress and severity of heart failure.

## Objectives

To assess whether plasma NT-PRO-BNPs kinetic could predict postoperative early outcome of CS [1].

## Methods

A prospective, descriptive study for 4 months (period: Oct 2014 - Jan 2015) in patients (pts.) admitted to our ICU. We included adults undergoing emergent, elective or semi-urgent heart valve replacement, coronary artery bypass grafting, and aortic aneurysm surgery. The pts. with chronic diseases that can increase the baseline NT-PRO-BNP level like chronic kidney disease, peripheral vascular disease, and chronic pulmonary disease, were excluded. Data, including perioperative and outcome data, were collected daily for 5 days. The postoperative complications were reflected by inotropic and vasoactive drugs used, postoperative acute renal failure, and mechanical ventilation time longer than 24 hours. The results were reported as median (interquartile range).

## Results

A total of 37 pts. (75,7% males), of 84 pts. admitted to ICU for postoperative care after CS, were included. Mean age was 65 ± 12 years. APACHE II on ICU admission was 26 ± 3. Mean ICU stay was 3 ± 1,6 days. NT-PRO-BNP concentration increased from 703 (5,3-3403) preoperatively to peak level of 1731 pg/ml (276-6674), reached at 48 hours postoperatively. Serum NT-PRO-BNP peak value in complicated vs. non-complicated CS was 1847 (50,2-6674) vs. 1372 (960-1965) pg/ml (p = 0,003). Pts. who developed postoperative acute renal failure (n = 12) had preoperative NT-PRO-BNP of 1132 (33,5-3403), vs. 496 (5,3-3263) pg/ml registered in pts. with preserved renal function (p = 0,005). Preoperative serum NT-PRO-BNP in pts. who needed for inotropic and vasoactive suport (n = 23) was 867 (5,3-3400), vs. 432 (33,5-1191) pg/ml in pts. who did not require perioperative inotropic and vasoactive medication (p = 0,003). No correlation between time of mechanical ventilation, ICU lenght of stay, and preoperative NT-PRO-BNP level were founded. Decreased preoperative left ventricular ejection fraction was detected in 29,7% pts. In those cases we founded postoperative serum NT-PRO-BNP peak level of 3034 (747-7550), vs. 1394 (154-4454) pg/ml in pts. with preserved preoperative ventricular function (p = 0,001).

## Conclusions

Preoperative NT-PRO-BNP is a valuable marker in predicting early postoperative outcomes after CS. High preoperative NT-PRO-BNP levels were associated with the need for inotropic and vasoactive support, and the deterioration of renal function in the early postoperative period. The peak levels, reached at 48 hours postoperatively, correlated with preoperative ejection fraction.
